# Secreted *Listeria* adhesion protein (Lap) influences Lap-mediated *Listeria monocytogenes* paracellular translocation through epithelial barrier

**DOI:** 10.1186/1757-4749-5-16

**Published:** 2013-06-24

**Authors:** Hyochin Kim, Arun K Bhunia

**Affiliations:** 1 Department of Food Science, Molecular Food Microbiology Laboratory, West Lafayette, USA; 2 Department of Comparative Pathobiology, Purdue University, West Lafayette, IN 47907, USA

**Keywords:** Listeria adhesion protein, Lap, *Listeria monocytogenes*, Secretion, Paracellular translocation, Pathogenesis

## Abstract

**Background:**

Listeria adhesion protein (Lap), an alcohol acetaldehyde dehydrogenase (*lmo1634*) promotes bacterial paracellular translocation through epithelial cell junctions during gastrointestinal phase of infection. Secreted Lap is critical for pathogenesis and is mediated by SecA2 system; however, if strain dependent variation in Lap secretion would affect *L. monocytogenes* paracellular translocation through epithelial barrier is unknown.

**Methods:**

Amounts of Lap secretion were examined in clinical isolates of *L. monocytogenes* by cell fractionation analysis using Western blot. Quantitative reverse transcriptase PCR (qRT-PCR) was used to verify protein expression profiles. Adhesion and invasion of isolates were analyzed by *in vitro* Caco-2 cell culture model and paracellular translocation was determined using a trans-well model pre-seeded with Caco-2 cells.

**Results:**

Western blot revealed that expression of Lap in whole cell preparation of isolates was very similar; however, cell fractionation analysis indicated variable Lap secretion among isolates. The strains showing high Lap secretion in supernatant exhibited significantly higher adhesion (3.4 - 4.8% vs 1.5 - 2.3%, *P* < 0.05), invasion and paracellular translocation in Caco-2 cells than the low secreting isolates. In cell wall fraction, Lap level was mostly uniform for both groups, while Lap accumulated in cytosol in low secreting strains indicating that Lap distribution in cellular compartments is a strain-dependent phenomenon, which may be controlled by the protein transport system, SecA2. Δ*secA2* mutants showed significantly reduced paracellular translocation through epithelial barrier (0.48 ± 0.01 vs 0.24 ± 0.02, *P* < 0.05). qRT-PCR did not show any discernible variation in *lap* transcript levels in either high or low secreting isolates.

**Conclusion:**

This study revealed that secreted Lap is an important determinant in Lap-mediated *L. monocytogenes* translocation through paracellular route and may serve as an indicator for pathogenic potential of an isolate.

## Background

Gastrointestinal (GI) phase of *Listeria monocytogenes* infection is complex and successful extra-intestinal dissemination of the pathogen from the GI tract to liver, spleen, gall bladder, central nervous system and the placenta (in case of pregnant women) is essential for systemic disease, listeriosis. During the early stage of infection, *L. monocytogenes* interacts with host intestinal cells. Thus, understanding this interphase between host and bacteria may aid in developing preventive or therapeutic strategies against listeriosis. Several virulence factors are responsible for initial interaction with the host. Among those, Internalin family of proteins including InlA and InlB play important roles during infection [1,2]. InlA interacts with E-cadherin and InlB with c-Met as host receptors during *Listeria* infection. Additionally, adhesion- and invasion-associated proteins including autolysin amidase (Ami), virulence invasion protein (Vip), fibronectin binding protein, LapB, InlJ, CtapB and several others are involved [3]. During intestinal phase, listerial survival under various intestinal environments such as acids, biles, antimicrobial peptides, mucus and resident microflora and their metabolites is critical and a majority of the stress response genes are regulated by Sigma B and/or PrfA [4,5].

Previously, our group showed that *Listeria* adhesion protein (Lap), a 104-kDa alcohol acetaldehyde dehydrogenase (*lmo1634*), is responsible for bacterial adhesion and transepithelial translocation through epithelial barrier [6-8]. Lap uses the host heat shock protein 60 (Hsp60) as a receptor [9], and the Lap-mediated *Listeria* adhesion is severely impaired in epithelial cells when *hsp60* gene is partially silenced by shRNA [7]. Cytosolic Lap is secreted to extracellular milieu with the help of the auxiliary secretion system, SecA2 [6,10]. Although both pathogenic and nonpathogenic *Listeria* species express Lap, only in pathogenic *Listeria*, secreted Lap is re-associated on the bacterial cell surface through an unknown mechanism, promoting *Listeria* interaction with the host cell [8]. Thus, we wanted to investigate the relationship between amounts of secreted Lap and the ability of the strain to adhere and translocate through epithelial cells among clinical isolates of *L. monocytogenes*. We examined the amounts of secreted Lap from different clinical isolates and determined cellular localization of Lap in the intracellular (cytosolic), cell wall and supernatant fractions among different isolates. Protein expression data were verified by *lap*-specific transcript (mRNA) analysis. Finally, the adhesion, invasion and paracellular translocation properties of these isolates were determined in an *in vitro* cell culture model.

## Materials and methods

### Bacterial cultures and growth conditions

Fifty six *L. monocytogenes* isolates were used in this study (Table 1) [11,12]. All *L. monocytogenes* isolates were grown in Tryptic soy broth with 0.6% yeast extract (TSBYE, Becton Dickinson) at 37°C. KB208 (*lap-*) was grown in the presence of erythromycin (5 μg/ml) at 42°C, and a *lap* complemented CKB208 (*lap*^*+*^) in TSBYE containing erythromycin (5 μg/ml) and chloramphenicol (5 μg/ml) at 37°C. SecA2 deletion mutant AKB103 (Δs*ecA2*) and the complemented strain AKB104 (*secA2*^*+*^) were grown in the presence of erythromycin (10 μg/ml) [6], while the internalin A deletion mutant AKB301 (Δ*inlA*) and the complemented strain AKB302 (*inlA+*) in presence of chloramphenicol (5 μg/ml) [7]. *L. monocytogenes* F4244 (WT), KB208 (*lap*-), CKB208 (*lap*+), AKB302 (Δ*inlA*) and AKB303 (*inlA*+) strains were used as controls as appropriate.

**Table 1:** **List of *Listeria monocytogenes *isolates used in this study**

**Culture**	**Serotype**	**Genotype**	**DUP no**	**Source**^**a**^
Clinical isolates				
F4244	4b	WT	DUP-1044	CDC, Listeriosis patient, CSF
-KB208		*lap-*	Insertion mutant	[13]
-CKB208		*lap-lap+*	Complemented strain	[13]
-AKB103		∆*secA2*	Deletion mutant	[6]
-AKB104		∆*secA2 secA2+*	Complemented strain	[6]
-AKB301		∆*inlA*	Deletion mutant	[7]
-AKB302		∆*inlA inlA+*	Complemented strain	[7]
V7	1/2a		DUP-1039	USFDA, Ohio outbreak, raw milk and cheese
Scott A	4b		DUP-1042	USFDA, Massachusetts milk outbreak, human, CSF
F4263	1/2b		DUP-1042	CDC, Listeriosis patient, CSF
F4243	4b		ND	CDC, Listeriosis patient, CSF
F4264	4b		DUP-1038	CDC, Listeriosis patient, CSF/Blood
F4262	4b		DUP-1051	CDC, Listeriosis patient, CSF/Blood
ATCC7644	1/2c		DUP-1039	ATCC, human
CHLR1	1		DUP-1023	CHLR, Blood, newborn female, 1984
CHLR2	1		DUP-1042	CHLR,CSF, two-week old female, meningitis, 1984
CHLR3	4		DUP-1038	CHLR, CAP isolate from CSF, human
CHLR4	1		DUP-1042	CHLR, CAP isolate from CSF; human
CHLR5	4		DUP-1042	CHLR, CSF, 11 day-old male, meningitis
CHLR6	1		DUP-1042	CHLR, female, meningitis
CHLR7	4		DUP-1024	CHLR, meningitis, child
CHLR8	1		DUP-1053	CHLR, 14-day old female, meningitis, 1985
CHLR9	4		DUP-1038	CHLR, CAP isolate from CSF, Human
CHLR10	4		DUP-1038	CHLR, 23-day old female, meningitis
CHLR11	1		DUP-1052	CHLR, 7-month old female, meningitis,1988
CHLR12	1		DUP-1053	CHLR, 11-day old male, meningitis,1988
CHLR1250	1		DUP-1048	CHLR, Blood, 11 year old male
V1	½ a		ND	Guinea pig lymph node
V2	½ c		DUP-1039	CSF human
V3	3a		DUP-1030	Human
V4	4a		DUP-1059	Sheep brain
V5	4b		DUP-1042	CSF, human
ATCC 19117	4d		DUP-1042	Human
H1	4b		DUP-1038	Aorta prothesis (69 years old patient), Sao Paulo, Brazil; 2001
H2	4b		DUP-18604	CSL (73 years old patient), Sao Paulo, 2000
H3	4b		DUP-19191	CSL (60 years old patient), Sao Paulo, 1998
H4	4b		DUP-1042	Blood (34 years old patient), Sao Paulo, 1998
H5	1/2a		DUP-1023	CSL (71 years old patient), Sao Paulo, 1995
H6	4b		DUP-18604	CSL (26 years old patient, HIV + death), Sao Paulo, 2001
H7	4b		DUP-1042	Blood (5 days old patient), Sao Paulo, 2004
H8	4b		DUP-1042	Blood (61 years old patient), Sao Paulo, 2003
H9	4b		DUP-1038	Blood (48 years old patient, death), Sao Paulo, 2004
H10	1/2a		DUP-19173	CSLF (55 years old patient), Sao Paulo, 2004
H11	1/2b		DUP-19175	Blood (72 years old patient), Sao Paulo, 2005
H12	4b		DUP-1038	CSL (death), Sao Paulo, 2005
H13	ND		DUP-1042	CSLF (16 years old patient), Sao Paulo, 2002
H14	ND		DUP-1042	CSL, Sao Paulo, 2002
H15	ND		ND	CSL, Sao Paulo, 2005
H16	ND		ND	Blood (6 days old patient), Sao Paulo, 1998
H14	ND		DUP-1042	CSL, Sao Paulo, 2002
H15	ND		ND	CSL, Sao Paulo, 2005
H16	ND		ND	Blood (6 days old patient), Sao Paulo, 1998
Food Isolates				
B30	B30	B30	B30	B30
E16	E16	E16	E16	E16
G21	G21	G21	G21	G21
T1	T1	T1	T1	T1
BB8	BB8	BB8	BB8	BB8
LL1	1		DUP-1044	Beef steak
F1	1		DUP-1029	Skinless boneless Chicken
H6	1		DUP-1039	Skinless boneless Chicken
GG8	1		DUP-1042	Skinless boneless Chicken
J1	1		DUP-1052	Skinless boneless Chicken
L32	4		DUP1044	Skinless boneless Chicken
S2	1		DUP1052	Skinless boneless Chicken
V6	4c		DUP-1061	VICAM, poultry
F1057	4b		DUP-1044	CDC, raw milk

^a^Note: *CSF* Cerebrospinal fluid; *ATCC* American Type Culture Collection; *CHLR* Children’s Hospital at Little Rock, Arkansas, USA; Isolates H1-H16 were originally isolated from Sao Paulo, Brazil [11]; *ND* Not determined.

### Protein secretion profiles and Western blot analysis

Bacterial protein fractionation method was used as described previously [6]. Briefly, 200 ml bacterial cultures were grown in TSBYE for 16 h at 37°C and centrifuged (7,000 × g, 10 min). The bacterial culture supernatants were filtered (0.22 μm) and trichloroacetic acid (final concentration 10%) was added for protein (SN) precipitation. The bacterial cell pellets were used for cell wall (CW) and intracellular (IN) protein preparation. After an overnight incubation at 4°C, the supernatants were centrifuged (14,000 × g for 10 min) at 4°C. Pellets were washed with ice-cold acetone and resuspended in alkaline rehydration buffer (100 mM Tris-base, pH 11, 3% SDS, and 3 mM DTT). For CW proteins, the bacterial pellet was resuspended and incubated with cell wall extraction buffer (10 mM Tri-HCl, pH 6.9, 0.5% SDS) for 30 min at 37°C. After centrifugation (14,000 × g for 10 min at 4°C), supernatants were collected as CW protein and the cell pellet was used for IN preparation. The pellets were suspended with 200 μl SDS-PAGE sample solvent and sonicated for 1 min (3 cycles of 20 min each). Samples were centrifuged (14,000 × g for 20 min) at 4°C and the supernatants collected.

For Western blot, SN and IN protein concentrations were measured using BCA assay (Peirce, Rockford, IL), while CW protein concentration was measured using Bradford assay (Bio-Rad). Each protein preparation (40 μg/well of SN, 20 μg/well of CW and IN) was separated by SDS-PAGE (7.5% or 10% -acrylamide) gel, and transferred to Immobilon-P membrane. Anti-Lap monoclonal antibody (MAb-H7), anti-NamA antibody (MAb-C11E9), anti-InlA PAb [7] were used as primary antibodies. Horseradish peroxidase conjugated secondary antibody (Jackson Immuno Research, West Grove, PA) and chemiluminescence system (Fisher Scientific, Pittsburgh, PA) were used for detecting protein bands. Densitometric analysis of potein bands was done by using Bio-Rad image analysis software.

An aminopeptidase C (PepC) assay was used to test SN and CW fractions for any contamination with cytosolic proteins [14]. Briefly, 10 μl of SN and CW proteins were added to 190 μl of 20 mM Tris–HCl (pH 7.4) and 2 μl of 200 mM L-arginine-p-nitroanilide (Sigma) in the same buffer and mixed. Five units of aminopeptidase C (Sigma) were used as positive control. Color change was measured at 405 nm for 10 min using a plate reader (Bio-Rad).

### qRT-PCR

Overnight grown select cultures were diluted 1:10 in 50 mL TSB and incubated for 4 h and bacterial RNA was extracted using Trizol method following RNase-free DNase I treatment (Fisher Scientific). The cDNA was synthesized from RNA (1 μg) using the iScript Reverse Transcriptase Kit (Bio-Rad), and 50 ng of cDNA was used as template for qRT-PCR using power SYBR Green (Applied Biosystem, Carlsbad, CA). Amplification of cDNA for *lap*, *inlA* and 16S rRNA was performed using the primers listed in Additional file 1: Table S1 at annealing temperature of 60°C for 40 cycles in StepOne™ real-time PCR system (Applied Biosystems). Each sample was run in triplicate. The relative expression levels of *lap* and *inlA* were calculated by ΔCt value using 16S rRNA as reference.

### Mammalian cell culture

A colon carcinoma cell line, Caco-2 (HTB37; ATCC) was grown in Dulbecco’s Modified Eagle Medium (Invitrogen, Grand Island, NY) containing 10% fetal bovine serum (D10F) (Atlanta Biologicals, Norcross, GA) at 37°C under 7% CO_2_ in a humidified incubator. Cells (passage 25–35) were seeded at approximately 5 × 10^4^ cells/well into 12 well plates (Corning) for adhesion or invasion assays, or 12 well transwell plate insert for transepithelial translocation (4 μm, Corning) experiment, and cell monolayers were used between 10 and 14 days.

### Adhesion and invasion analysis

Adhesion and invasion assays were conducted as described previously [6]. For adhesion analysis, bacterial cultures were added to Caco-2 cell monolayers at an MOI of 10:1 (bacteria: Caco-2 cells). After 1 h infection, cells were treated with Triton X-100 (0.1% v/v) and adherent bacteria were enumerated by plating on TSA-YE agar. To assess invasion, Caco-2 cells were infected for 1 h, and then treated with D10F containing 50 μg/ml gentamicin for additional 1 h to kill non-invaded bacteria. Invaded bacteria were enumerated on TSBYE agar plate. Each experiment was run at least three times in triplicate.

### Paracellular translocation assay

Transepithelial translocation experiments were performed as described previously [7]. Briefly, Caco-2 cells were grown as monolayers on Transwell Filter Inserts (pore size 4 μm; Corning). Transepithelial electrical resistance (TEER) of polarized monolayers was measured using a Voltmeter (Millipore). A minimum TEER of about 200 Ω/cm^2^ (200 ± 10) was required for each translocation experiment. Bacteria (MOI ~10:1) were added to the apical well of the transwell insert, and incubated at 37°C in 7% CO_2_ incubator for 2 h. Subsequently, the liquid was collected from the bottom well and translocated bacteria were enumerated by plating. Data are presented as percentage of bacterial cells that translocated through the cell junctions.

Tight junction integrity was determined by TEER reduction and FITC-dextran (4 kDa; Sigma) permeability [15]. To measure dextran permeability, 100 μl of 5 mg/ml FITC-dextran was added to apical side of the trans-well inserts. After an appropriate incubation time, translocated dextran was quantified by measuring emission at 485 nm and excitation at 520 nm using a spectrofluorometer (Molecular Devices LLC, Sunnyvale, CA).

### Statistical analysis

The Generalized Linear Model (GLM) procedure of SAS (SAS Institute, Cary, NC) was used to compare the differences in means. A P-value < 0.05 was considered to be significantly different for comparison between two treatments using Students *t*-test. Difference among the treatments was also determined using Tukey’s test.

## Results

### Protein secretion profiles and Western blot analysis show variable LAP secretion

**Figure 1:**
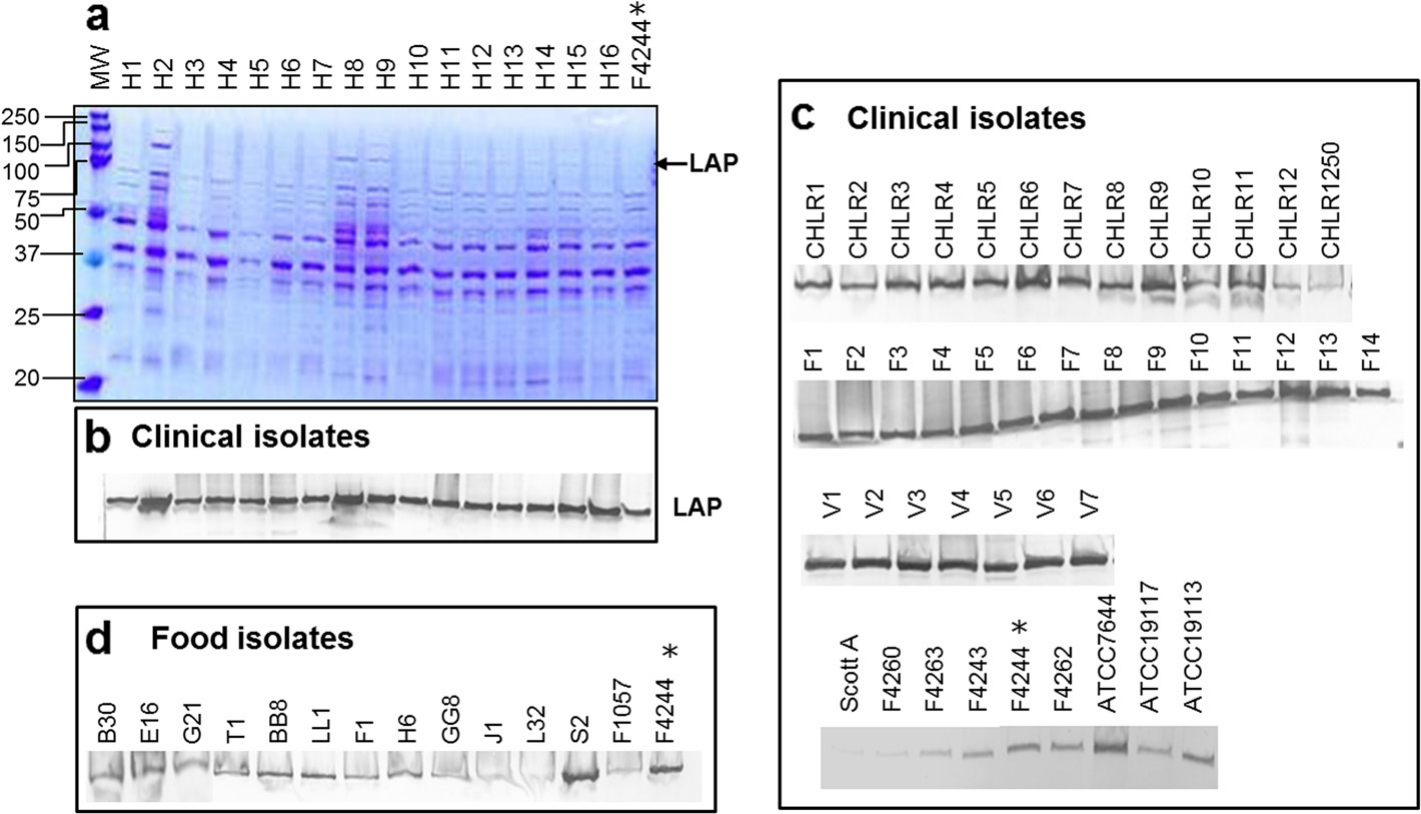
**SDS-PAGE and Western blot analysis of total Lap (without supernatant fraction) *L. monocytogenes *isolates.** Total protein was extracted from whole cells using sample solvent buffer and sonication (3 cycles of 20 min each). Each well was loaded with 5 μg protein in SDS-PAGE (4-15% polyacrylamide) gel. (**a**) Coomassie blue stained gel, (**b**) Corresponding Western blot immunoprobed with anti-Lap antibody (MAb-H7), Western blot of additional (**c**) clinical isolates and (**d**) food isolates. *F4244 is used as a positive control.

Initially, we examined LAP expression in protein preparations from whole cells (excluding supernatant fractions) of 47 clinical isolates and 9 food isolates of *L. monocytogenes* from our culture collection (Table 1) and all were positive for Lap. A representative Coomassie blue stained gel (Figure 1a) verified uniform protein loading amounts in gel prior to performing Western blot analysis for a set of clinical isolates. We observed uniform amounts in most isolates with a minor variation in some clinical isolates (Figure 1b, 1c). While the intensity of Lap band in total protein extracts from food isolates, in general, weaker than the most clinical isolates (Figure 1d), its significance in pathogenesis was not explored. In subsequent experiments, we chose a set of clinical isolates (F4244, H4, H9 and H13), which appeared to have slightly higher amounts of total Lap (based on band intensity) than a set (ATCC 19117, F4263, CHLR4 and CHLR6) with lower amounts and examined for Lap distribution in different cell fractions (supernatant, cell wall and intracellular).

**Figure 2:**
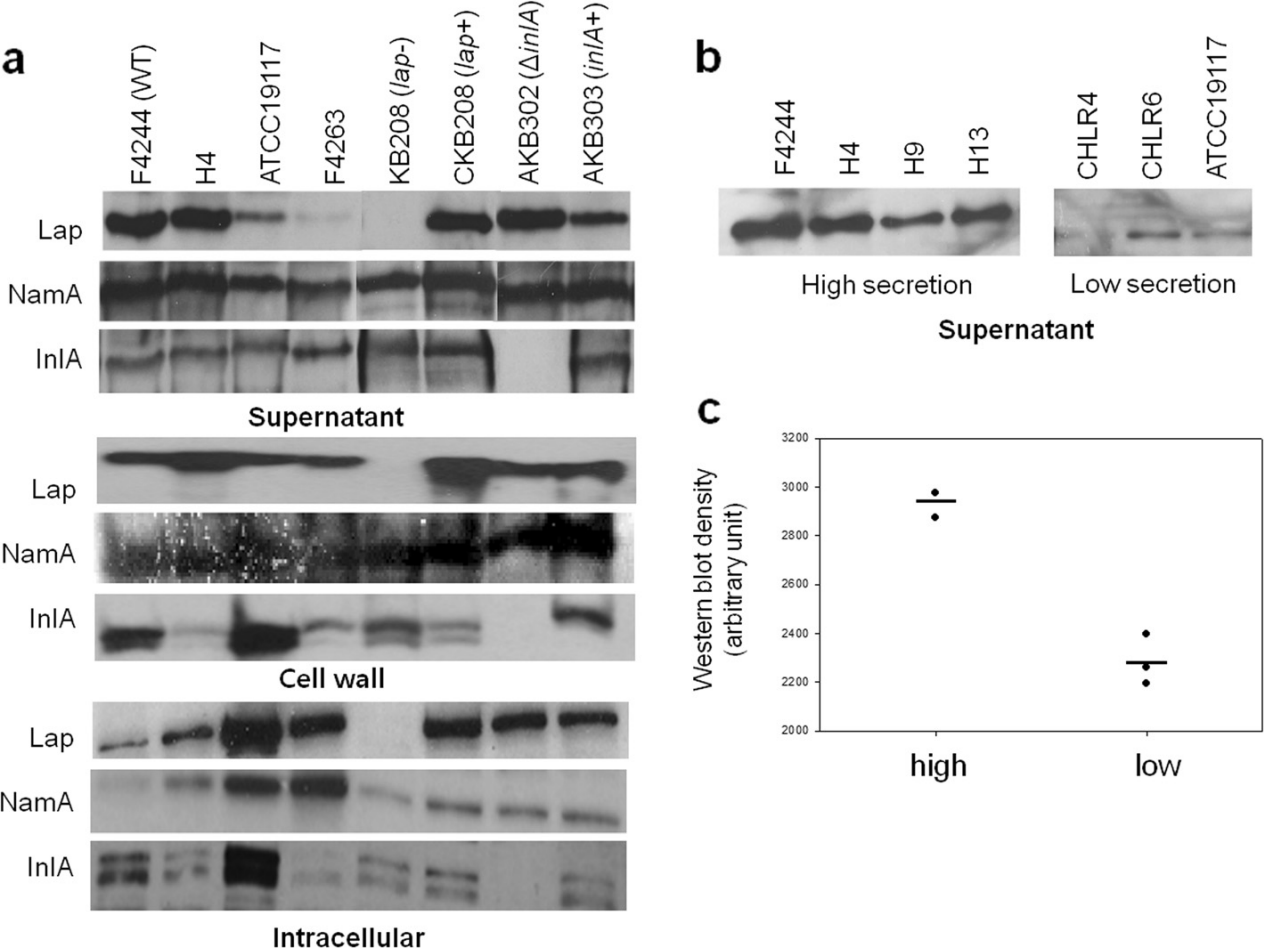
**Analysis of Lap distribution in cellular fractions.** (**a**) Western blot analysis showing levels of Lap in different cell fractions (supernatant, cell wall and intracellular). Distribution of N-acetyl muramidase (NamA) and InternalinA (InlA) was also monitoted in the same cell fractions. (**b**) Lap secertion levels in supernatant of several other clinical isolates (H4, H9, H13, CHLR4, CHLR6) were monitored. (**c**) Densitometric analysis of intesity of Lap bands from panel 2b. *L. monocytogenes* F4244 (WT), *lap* mutant (*lap*-), *lap* complemented (*lap*+), Δ*inlA* and *inlA* complemented (*inlA*+) strains were used as controls.

Cell fractionation study revealed that among the isolates tested, F4244, H4, H9 and H13 strains secreted a large amounts of Lap in the supernatant fraction (Figure 2a) while ATCC19117, F4263, CHLR4, and CHLR6 had low to negligible amounts of secreted Lap (Figure 2b) as verified by densitometric analysis (Figure 2c). In the cell wall fraction, the protein levels were very similar for both high (F4244, H4) and low Lap secreting isolates (ATCC19117, F4263); however, in the intracellular (cytosolic) fraction, high secreting isolates had the least amount of Lap while the low secreting strains had high amounts. Prior to Western blot analysis, PepC assay performed with the supernatant and cell wall fractions did not indicate any contamination with the intracellular proteins (Additional file 2: Figure S1). We also examined Lap secretion level in *lap*^*-*^ (KB208) and Δ*inlA* mutants and their respective complemented strains, and data were in agreement with our previous observation [6]. The levels of secreted InlA in supernatant were uniform for all the isolates tested except for the Δ*inlA*; however, its localization in cell wall and the intracellular fraction was variable in both high and low Lap secreting strains (Figure 2a). As a control, we also examined the secretion of NamA, a well-recognized secreted protein in *Listeria*, whose level was uniform in both high and low Lap secreting *L. monocytogenes* isolates.

### *lap* transcripts level

**Figure 3:**
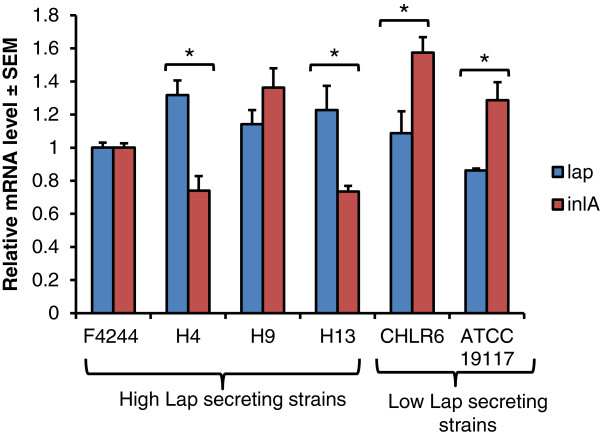
**Lap transcript level in high and low Lap-secreting isolates.** mRNA levels for *lap* and *inlA* from high and low Lap-secreting isolates were determined from 18-h grown cultures. 16S was used as an internal control. Data are average of two independent experiments performed in triplicate (n ≥ 6). * denotes significant difference between *lap* and *inlA* transcript levels in a strain at *P* < 0.05.

We examined *lap* transcript level in select high and low Lap secreting isolates using qRT-PCR and data were compared with *inlA* as a positive control and 16S rRNA as an internal control. Levels of *lap* transcripts in both high and low secreting isolates were very similar (Figure 3), which is in agreement with Western blot data showing similar amounts of total Lap. Furthermore, we also observed that among the high Lap secreting isolates (H4 and H13), *lap* transcript levels were significantly (*P* < 0.05) higher than the *inlA* transcripts while, *inlA* was higher for low *lap*-secreting isolates (CHLR6 and ATCC19117).

### Adhesion and invasion characteristics of high and low LAP secreting isolates

**Figure 4:**
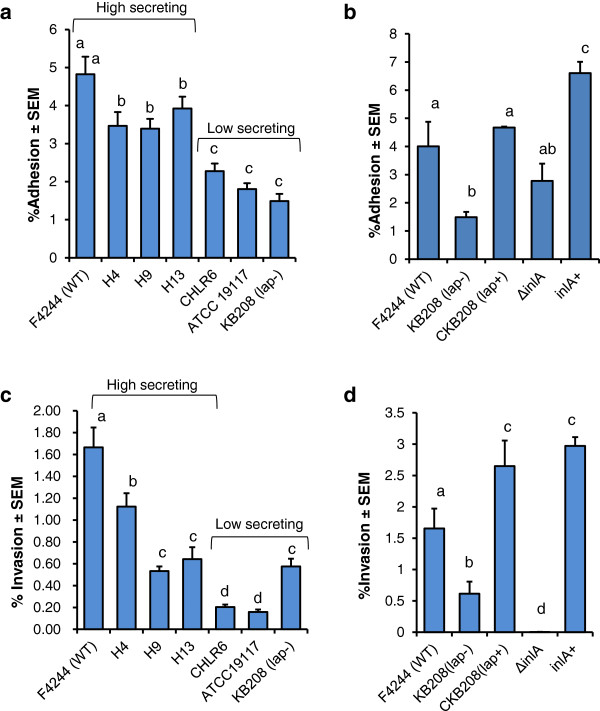
**Adhesion and invasion properties of *L. monocytogenes.*** (**a** and **b**) Adhesion and (**c** and **d**) invasion characteristics of high and low-Lap secreting isolates analyzed in Caco-2 cell culture model. F4244 (WT), *lap-* mutant (KB208), Δ*inlA* and *inlA* complemented (*inlA*+) strains were used as controls. Data are averages of at least three independent experiments performed in triplicate (n ≥ 9). Bars marked with different letters (a,b,c,d,) are significantly different at *P* < 0.05.

Adhesion characteristics of *L. monocytogenes* strains (H4, H9, and H13) showing high levels of secreted Lap were significantly (*P* < 0.05) higher than that of low secreting isolates (CHLR6, ATCC 19117). Adhesion data were comparable to our wild type F4244 for high secreting and the *lap*-deficient KB208 for low secreting strains (Figure 4a, 4b).

We also examined the invasive properties of these isolates. In general high Lap secreting isolates (F4244, H4, H9, and H13) invaded Caco-2 cells at a higher percentage than the low LAP secreting strains (CHLR6, ATCC 19117, KB208) (Figure 4c, 4d). As expected, adhesion (Figure 4b) and invasion (Figure 4d) profiles of Δ*inlA* and the complemented strain (*inlA*+) were in agreement with previous observation [16]. Even though Lap is not directly responsible for *L. monocytogenes* invasion [6,8], it has been demonstrated earlier that increased adhesion facilitates increased contact of bacteria with epithelial cells hence promoting increased invasion by *L. monocytogenes* by utilizing invasion-associated proteins such as InlA, InlB, Vip, and Auto, and others [3,17].

### High Lap secreting isolates show increased paracellular translocation

**Figure 5:**
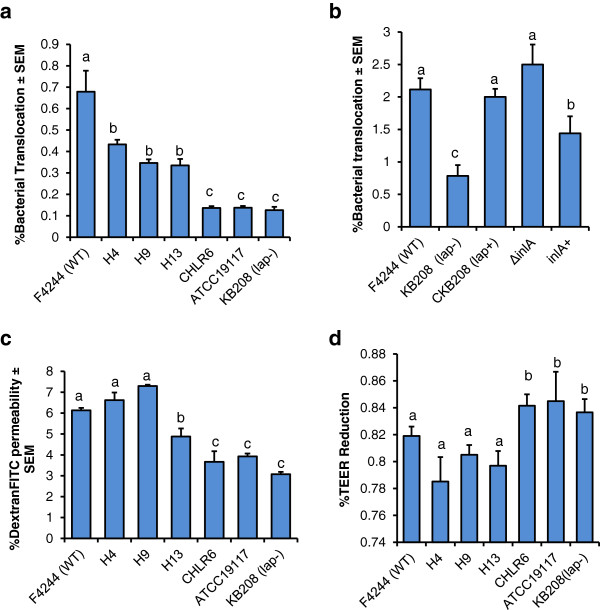
**Transepithelial translocation characteristics of *L. monocytogenes.*** (**a** and **b**) Transepithelial translocation characteristics of *L. monocytogenes* through paracellular route in Caco-2 cell monolayers by trans-well assays. (**c**) Dextran^FITC^ permeability through Caco-2 cell monolayer in trans-well model and (**d**) Transepithelial electrical resistance (TEER) in Caco-2 cell monolayers after treatment with high (H4, H8, H13) and low (CHLR4, CHLR6, ATCC 19117) Lap secreting isolates. *L. monocytogenes* F4244 (WT), *lap* mutant (*lap*-), *lap* complemented (*lap*+), Δ*inlA* and *inlA* complemented (*inlA*+) strains were used as controls. Data are average of at least three independent experiments performed in triplicate (n ≥ 9). Bars marked with different letters (a,b,c) are significantly different at *P* < 0.05.

Previously, we showed that Lap promotes *L. monocytogenes* translocation through epithelial barrier using paracellular route [7]. Thus, we examined paracellular translocation characteristics of high and low Lap secreting strains through epithelial barrier in a well-established trans-well experiment. Data show that the high Lap-secreting isolates translocated through epithelial barrier at significantly higher frequency (*P* < 0.05) than the low Lap-secreting isolates (Figure 5a). The *lap* mutant (KB208) showed significantly reduced translocation; however, Lap-mediated translocation was restored in a *lap*-complemented strain (CKB208). This result clearly indicates that secreted Lap is critical for bacterial translocation through epithelial cell barrier. Furthermore, as a control, we also examined the translocation of Δ*inlA*, and this strain showed a very high percentage (0.21 ± 0.02) of translocation similar to the WT (0.25 ± 0.03) (Figure 5b) and these data are in agreement with our previous observation [7]. Increased paracellular translocation through epithelial barrier by Δ*inlA* is speculated to be a compensatory mechanism for the strain since it is unable to invade mammalian cell through intracellular route thus producing a higher amount of Lap (Figure 1a).

Increased paracellular translocation is proposed to be facilitated by Lap-mediated compromise of the epithelial barrier [7] and the cellular mechanism of tight junction compromise is still under investigation. Lap-induced epithelial-barrier compromise was monitored by assaying Dextran^FITC^ permeability from apical to basolateral side of the cell monolayer in the trans-well insert. Data again showed that the high Lap-secreting strains have significantly higher (*P* < 0.05) dextran permeability than the low Lap-secreting strains (Figure 5c). Furthermore, the high Lap-secreting strains also significantly have reduced TEER compared to low secreting isolates (Figure 5d). These data clearly show that secreted Lap is critical in Lap-mediated cell-cell junction compromise and promotion of *L. monocytogenes* paracellular translocation through epithelial barrier.

**Figure 6:**
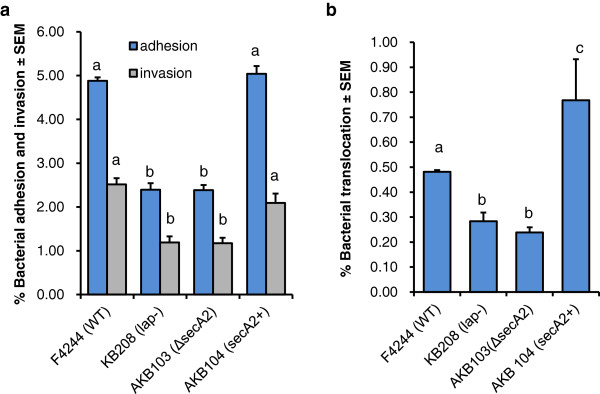
**Involvement of SecA2 in adhesion, invasion, and translocation in *L. monocytogenes.*** Analysis of involvement of SecA2 in *L. monocytogenes* (**a**) adhesion and invasion, and (**b**) translocation through paracellular route in Caco-2 cell monolayers in trans-well assay using WT strain F4244, *secA2* mutant (Δ*secA2*) and *secA2* complemented strain (*secA2*+). Data are average of at least three independent experiments performed in triplicate. Bars marked with different letters (a, b, c) are significantly different at *P* < 0.05.

Earlier, we had shown that the Lap secretion to extracellular milieu is mediated by an auxiliary secretion protein, SecA2 and deletion of *secA2* gene in F4244 affected bacterial adhesion and invasion (Figure 6a). Here, we showed that paracellular translocation was also severely impaired in a Δ*sec2* mutant but restored in the complemented (*secA2*+) strain (Figure 6b). We also observed similar trend in another strain of *L. monocytogenes*, 10403S (serovar 1/2a) and the paracellular translocation was significantly (*P* < 0.05) lower for Δ*secA2* deletion mutant (data not shown). Furthermore, the Δ*sec2* strain also exhibited reduced dextran permeability in Caco-2 cell trans-well insert model (data not shown). This study further confirms that Lap secretion is SecA2-dependent and impaired SecA2 affects Lap-mediated bacterial paracellular translocation.

## Discussion

*L. monocytogenes* is an opportunistic foodborne pathogen that infects primarily immunocompromised individuals such as the elderly, neonates, organ transplant patients or cancer patients receiving chemotherapy and pregnant women. Intestinal phase of listeriosis is complex and remarkably, *Listeria* has developed unique survival skill through expression of several virulence genes that ensure its survival in the presence of acids, enzymes, bile salts, mucus, antimicrobial peptides, resident microflora and their metabolites in the gut [4,5]. Crossing of intestinal epithelial barrier is a critical event for initiating systemic listeriosis. M cells [18] in Peyer’s patches and in solitary intestinal lymphoid tissue (SILT) and mucus secreting goblet cells [17], in part, facilitate, *L. monocytogenes* translocation. In addition, several virulence proteins play important role during bacterial dissemination through epithelial barrier [4]. Among these, InlA following interaction with E-cadherin promotes bacterial invasion and translocation through intracellular route. E-cadherin is a major protein in adherence junction (AJ) and is localized at the basolateral side of the cell junction; thus it is normally unavailable for interaction with InlA for luminal bacteria [17]. During epithelial necrotic cell extrusion process at the villus tip, InlA is able to interact with E-cadherin facilitating *L. monocytogenes* invasion [19]. Recently, we have shown that Lap, after interaction with epithelial Hsp60, increases tight junction permeability and promotes bacterial translocation through paracellular route [7]. During this paracellular translocation process, we hypothesize that *L. monocytogenes* will also have increased interaction with E-cadherin promoting listerial crossing of epithelial barrier by either InlA-dependent or InlA-independent pathway such as seen for the Δ*inlA*-mutant [7]. A Lap-positive but Δ*inlA*-mutant strain efficiently translocates through epithelial barrier in the trans-well model (Figure 5) reinforcing the notion that bacteria are indeed capable of translocating using the paracellular route [7] to cause systemic infection. It is speculated that deletion of *inlA* gene may favor *L. monocytogenes* to use the paracellular route to cross epithelial barrier as an alternative strategy for successful systemic spread.

In a previous experiment, surface Lap expressing *Lactobacillus paracasei* was able to cross epithelial barrier through paracellular route at a significantly higher percentage than the WT strain [20]. Furthermore, the Lap-expressing recombinant probiotic strain was also able to reduce *L. monocytogenes* translocation by 46% in a trans-well model emphasizing again the significance of Lap in bacterial paracellular translocation [20].

Lap is alcohol acetaldehyde dehydrogenase, a house keeping enzyme [8]. It is produced by both pathogenic and nonpathogenic *Listeria* and only in pathogenic species (*L. monocytogenes* and *L. ivanovii*); the secreted Lap re-associates on the surface using an unknown mechanism and promotes Lap-mediated adhesion and paracellular translocation. Surface re-association mechanism possibly is defective in nonpathogenic bacteria [8]. Since Lap plays a critical role during intestinal phase of infection and secreted Lap is an important determinant for Lap-mediated pathogenesis [6,8], it is relevant to determine if variation in Lap secretion among the clinical isolates would affect bacterial paracellular translocation. We tested 47 clinical isolates and 9 food isolates from our culture collection and the total Lap from whole cell preparations (except supernatant proteins) appears to be very similar for all the clinical strains tested. In general, food isolates have reduced level of total Lap compared to clinical isolates; however, importance of such findings is not pursued any further in these isolates.

In clinical isolates, Lap in the supernatant fraction was highly variable with some isolates secreting larger quantities than the others. Further cell fractionation analysis revealed that the high Lap-secreting isolates had very low levels of cytosolic Lap, while low secreting isolates accumulated higher amounts of cytosolic Lap (Figure 2). Cell wall-associated Lap remained mostly uniform for both groups and its involvement in Lap-mediated pathogenesis is not fully understood. This experiment indicates that total amount of Lap to be constant for both groups and only observable differences were in distribution of Lap among the cellular compartments. To verify if protein expression is in agreement with transcript (mRNA) level in cells, qRT-PCR was performed and data were in agreement with protein data indicating possible variation in protein secretion may be a strain-dependent phenomenon where protein translocation by SecA2 system is involved (see below). Interestingly, high Lap-secreting strains (observed only in H4 and H13) had significantly lower levels of *inlA* transcript while low Lap secreting strains had significantly higher amounts of *inlA* transcripts (Figure 3). This suggests a possible balance in virulence factor expression in pathogens may happen, which is quite intriguing and warrant further investigation.

We have shown earlier that Lap secretion to the extracellular milieu, but not to the cell wall, is facilitated by the SecA2 system [6,10]. We determined if reduced Lap secretion in certain isolates may be associated with impairment in SecA2 function in these isolates. To verify, we analyzed the secretion of N-acetyl muramidase (NamA), an autolytic enzyme, whose secretion is dependent on SecA2 [21]. Western blot analysis revealed that there was no difference in secreted (supernatant) and the cell wall associated NamA (Figure 2) in both high and low Lap secreting isolates indicating that SecA2 system is functional. However, protein (substrate)-dependent SecA2 transport mechanism cannot be ruled out in bacterial strains [22]. Involvement of SecA2 in Lap secretion was further verified by performing paracellular translocation experiment. SecA2 deletion mutant from two different strains (F4244 and 10403S) showed reduced paracellular translocation, providing a circumstantial evidence that impaired secretion of Lap possibly affecting bacterial paracellular translocation. It is important to note that SecA2 has been involved in translocation of many other virulence factors [21] and some of which may also contribute to bacterial translocation through epithelial barrier that cannot be ignored.

Lap being a house keeping enzyme [8], its sequence is highly conserved. Thus, variation in *lap* gene sequence in high and low Lap secreting isolates is not expected to be involved in strain dependent protein secretion. We compared the Lap sequence of our F4244 (WT) strain with published whole genome sequence from nine *L. monocytogenes* isolates in the NCBI database, and the sequences were found to be highly conserved among isolates (99.4-99.8% similarity) (data not shown) providing a circumstantial evidence that sequence variation may not be a contributory factor for variation in Lap secretion in isolates.

Since the secreted Lap is critical in pathogenesis [8], we examined if variable Lap secretion from clinical isolates from patients and some of whom suffered from fatal listeriosis, would exhibit differential pathogenic attributes such as adhesion, invasion and paracellular translocation. We observed that high Lap-secreting isolates exhibited significantly increased adhesion to, invasion into and paracellular translocation through Caco-2 cells compared to low Lap-secreting isolates. Even though the low Lap-secreting isolates translocated at a reduced level they still are able to cross the epithelial barrier, albeit at lower number, to cause systemic disease but severity of infection may be lower than the high Lap secreting isolates. Since we do not have detailed clinical history of patients from whom each of the isolates was isolated, it is difficult to validate our observation. However, in our previous mouse experiment, we have demonstrated that WT strain secreting increased amounts of Lap under anaerobic condition exhibited higher extra-intestinal dissemination to liver and spleen compared to the strain that was grown under aerobic condition secreting a reduced level of Lap [6]. Furthermore, high Lap-secreting isolates induced enhanced epithelial cell junction permeability as evidenced by increased dextran permeability through the cell monolayers than the low secreting strains (Figure 5). These findings strongly suggest that variation in Lap secretion by *L. monocytogenes* isolates could serve as a predictor of pathogenic potential and clinical outcome.

## Conclusion

We investigated the role of secreted Lap in listerial infection in an *in vitro* cell culture model. We show that isolates secreting increased amounts of Lap also exhibited increased paracellular translocation by inducing increased tight junction permeability compared to the low Lap-secreting isolates. Strain-dependent Lap secretion is speculated to be the function of the protein secretion system, SecA2 and the amount of secreted Lap may serve as a predictor for pathogenic potential in an isolate.

## Competing interest

We all declare that there are no competing interests.

## Authors’ contribution

AKB and HK designed the experiments and HK performed them. Both HK and AKB analyzed the data and wrote the manuscript. Both authors approved the manuscript for publication.

## Supplementary Material

Additional file 1: Table S1 List of primers used in this study. Click here for file

Additional file 2: Figure S1 Aminopeptidase C (PepC) assay of supernatant preparations did not indicate any Contamination with intracellular (cytosolic) proteins during protein preparation. Data taken from one representative experiment performed in triplicate (n = 3). Click here for file
