# Supplementary material for: Secreted Listeria adhesion protein (Lap) influences Lap-mediated Listeria monocytogenes paracellular translocation through epithelial barrier
Source: Gut Pathog. 2013 Jun 24;5:16. doi: 10.1186/1757-4749-5-16 (PMC3716925; doi:10.1186/1757-4749-5-16)
Supplement: Additional file 1: Table S1 — List of primers used in this study. [file 1757-4749-5-16-S1.pdf]

Table S1. List of primers used in this study

Gene	Primer	Oligonucleotide sequence (5'-3')	Source
**LAP (Full length)**	LAP Full F	GACCATGG*ATGGCAATTAAAGAAAATG	[Kim KP, 2004]
	LAP Full R	GACTCGAGAACACCTTTGTAAGCTT	
**LAP**	LAP qRT-PCR F	CCACAGCTTGGCACACAAAAA	this study
	LAP qRT-PCR R	CGCACGACCCGTGAAGGAA	
**InlA**	InlA qRT-PCR F	TGGCGCTTTGATTGCACCTGCTA	this study
	InlA qRT-PCR R	AGTGGCTGCGTCACGGTTCCA	
**16S**	16S F	GGTGCATTAGCTAGTTGG	
	16S R	AATCCGGACAACGCTTGC	

**Nco*I restriction enzyme site
