# Supplementary figures and images for: Secreted Listeria adhesion protein (Lap) influences Lap-mediated Listeria monocytogenes paracellular translocation through epithelial barrier
Source: Gut Pathog. 2013 Jun 24;5:16. doi: 10.1186/1757-4749-5-16 (PMC3716925; doi:10.1186/1757-4749-5-16)

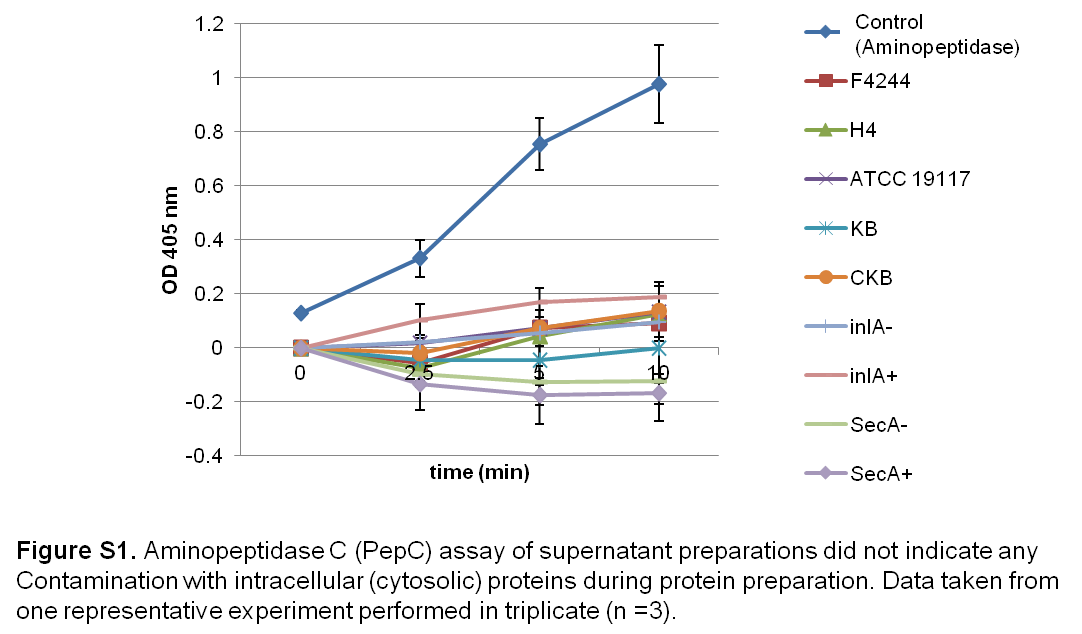

Supplement: Additional file 2: Figure S1 — Aminopeptidase C (PepC) assay of supernatant preparations did not indicate any Contamination with intracellular (cytosolic) proteins during protein preparation. Data taken from one representative experiment performed in triplicate (n = 3). [file 1757-4749-5-16-S2.png]
